# The sense of loneliness and meaning in life in post-COVID convalescents—a preliminary study

**DOI:** 10.3389/fpsyt.2023.1296385

**Published:** 2023-12-21

**Authors:** Kasper Sipowicz, Tadeusz Pietras, Anna Mosiołek, Michał Sobstyl, Michał Ring, Krystian Kamecki, Ignacy Stefańczyk, Marcin Kosmalski

**Affiliations:** ^1^Department of Interdisciplinary Disability Studies, The Maria Grzegorzewska University in Warsaw, Warsaw, Poland; ^2^The Second Department of Psychiatry, Institute of Psychiatry and Neurology in Warsaw, Warsaw, Poland; ^3^Department of Clinical Pharmacology, Medical University of Lodz, Lodz, Poland; ^4^Department of Forensic Psychiatry, Institute of Psychiatry and Neurology in Warsaw, Warsaw, Poland; ^5^Neurosurgery Department, Institute of Psychiatry and Neurology in Warsaw, Warsaw, Poland

**Keywords:** depression, SARS-CoV-2 infection, feeling of loneliness, sense of meaning in life, DJGLS, LAP-R

## Abstract

**Introduction:**

The COVID-19 epidemic has provided opportunity to study the impact of a well-defined severe illness on the development of a depressive episode and the associated sense of loneliness and lack of meaning in life.

**Materials and Methods:**

The aim of the study was to assess the occurrence of a reactive depressive episode, the severity of depression, a sense of loneliness and meaning in life in subjects who approximately a year earlier than the date of the study had suffered from a pulmonary form of SARS-CoV-2 infection with radiologically documented interstitial lesions of the lungs, requiring and not requiring hospitalization compared to people who did not develop the disease as a result of infection with that virus. The study included 63 subjects hospitalized for pulmonary lesions, 67 not hospitalized and 60 healthy controls. The severity of depressive symptoms was measured using a Polish-language standardized version of the Beck Depression Inventory, a sense of loneliness using the De Jong Gierveld Loneliness Scale, and a sense of meaning in life using the Life Attitude Profile-Revised.

**Results:**

The frequency of depression and its severity were found to be the highest in hospitalized patients compared to those treated at home and healthy people. A significant difference in the frequency of depression and its severity between outpatients and healthy people was also observed. The feeling of loneliness turned out to be greatest in the group of hospitalized people. Also, the severity of loneliness was found to be higher in the outpatient compared to the control group. The sense of meaning in life reached its lowest level among hospitalized patients, was moderately reduced in the outpatient group, and typical of the Polish population in the control group.

**Discussion:**

Both pulmonary SARS-CoV-2 infection and hospitalization have been shown to be a risk factor for depression, increased feeling of loneliness and a reduced sense of meaning in life. The effect of trauma and the presence of depression can be the explanation for the increased sense of loneliness after the illness and the partial breakdown of the lifeline manifested by a decrease in the sense of meaning in life.

## 1 Introduction

Surviving the SARS-CoV-2 pandemic was a completely new experience for humanity at the turn of the second and third decade of the twenty-first century (1). The last time humanity encountered a global epidemic of such intensity was 100 years ago during the Spanish flu epidemic, resulting in the death of more people than during the First World War (2, 3). The severe course of the disease in some patients with a fatal outcome, high incidence and lockdown were a strong stressor for many people (4, 5). Patients who required hospitalization due to pulmonary complications of the type of interstitial changes and due to the effects of thrombosis accompanying the infection were particularly vulnerable to severe stress (6). The need for hospitalization meant a severe course with a high degree of dyspnea, complete isolation from the family and exposure to the deaths of other hospitalized patients (7). This was probably accompanied by very strong fear for oneself and the loved ones, reactive depressed mood, and sometimes the development of full-blown type I PTSD (8). The mass nature of the pandemic, high fatality and mortality rates, as well as the low effectiveness of the implemented treatment could have reformulated the question about the meaning of life and the worldview in many people and could have disturbed their system of values and hierarchy of needs (9, 10). Those who recovered were exposed to severe psychological trauma caused by somatic suffering, isolation and loneliness, as well as significant psychological suffering (11, 12). Recovery should be treated as a success, but the effects of SARS-CoV-2 infection are still felt by these people (13). Convalescent patients complain of memory disorders, various respiratory problems and anxiety-depressive disorders (14, 15). It has been noted that during the COVID-19 pandemic, there has been an increase in feelings of fatigue, loneliness, anxiety and depression, dysfunctional coping strategies in different populations (including, among others, the general population and the population of health care workers) (16–20). The social isolation forced by the pandemic and the associated sense of loneliness is likely to have been the reason for the observed increase in the severity of anxiety, depression and the use of ineffective ways of coping with stress. However, this hypothesis, seemingly obvious, requires empirical verification.

It is interesting whether the effects of the SARS-CoV-2 experience differ in people who required hospitalization vs. those treated in home isolation and those who did not contract the infection during the pandemic.

The aim of the study is to compare the severity of loneliness, the severity of depressive symptoms and the value of dimensions of the sense of meaning in life between the hospitalized group, the group treated at home and people who did not develop SARS-CoV-2. In connection with the aim of the work, the following research questions were formulated:

1. Do convalescents hospitalized, non-hospitalized, and subjects who had no COVID infection differ in terms of:

a) Severity of depression;
b) Severity of the feeling of loneliness;
c) The obtained values of all LAP-R scale dimensions.

1. Do the results obtained with the LAP-R scale correlate with the severity of depression and feelings of loneliness?
2. Does the severity of depression correlate with a sense of loneliness and a sense of meaning in life?
3. Were there any differences between men and women observed?
4. Does the age of the respondents correlate with the studied variables?

## 2 Materials and methods

### 2.1 Subjects

The tests were carried out in the period 01.2022–06.2023 at the Outpatient Department of Allergology and Lung Diseases of the Norbert Barlicki Memorial University Teaching Hospital No. 1 of the Medical University of Łódź and at the primary care outpatient department in Aleksandrów Łódzki.

Originally, the study enrolled 105 people hospitalized due to SARS-CoV-2 infection, 110 people who had SARS-CoV-2 without hospitalization, and 92 people who had not been infected with SARS-CoV-2.

The criteria for exclusion from the study were as follows:

- no consent to participate in the study,
- the failure to complete all survey questionnaires,
- the occurrence of thromboembolism in a patient before contracting SARS-CoV-2,
- the presence of congenital thrombophilia and blood coagulation disorders confirmed by the patients’ medical records,
- past myocardial infarction or stroke before contracting SARS-CoV-2,
- the presence of malignancy within up to 5 years before contracting SARS-CoV-2,
- the confirmed presence of autoimmune diseases and congenital immune disorders,
- intellectual disability,
- dementia or active psychosis.

The information concerning the exclusion criteria came both from medical histories and the data obtained from patients. The low median age of subjects enrolled in our study is due to the high co-morbidity of the elderly patients.

One hundred and sixty patients were included in the study, including 63 convalescents hospitalized due to SARS-CoV-2 infection, 67 convalescents who did not require hospitalization due to SARS-CoV-2 infection and 60 subjects who did not have SARS-CoV-2 infection. The median age for all patients was 48.00 (Q1–Q3: 39.00–61.00) and did not differ with statistical significance between the groups (*p* = 0.754). All patients in the hospitalized group of convalescents required treatment with oxygen therapy or a respirator while in the other two groups none of the patients required oxygen therapy. Patient demographics are presented in Table 1.

**Table 1:** Demographic data of patients participating in the study.

Variable		Convalescents who were hospitalized due to SARS-CoV-2 infection (*N* = 63)	Convalescents who did not require hospitalization due to SARS-CoV-2 infection (*N* = 67)	Patients who have never had a SARS-CoV-2 infection (*N* = 60)
Sex	Female	35 (55.56%)	32 (47.76%)	31 (51.67%)
	Male	28 (44.44%)	35 (52.24%)	29 (48.33%)
Age (21)		48.00 (39.00–63.00); Min-Max: 30.00–75.00	47.00 (39.00–60.00); Min-Max: 30.00–76.00	49.50 (39.50–61.50); Min-Max: 30.00–75.00
Oxygen therapy/respirator	Yes	63 (100%)	0.00 (0%)	0.00 (0%)
	No	0.00 (0%)	67 (100%)	60 (100%)

The place of residence of the patients was not analyzed, as both the family doctor’s clinic in Aleksandrów Łódzki and the N. Barlicki Memorial hospital are located in the metropolitan districts of the Łódź agglomeration, which is inhabited by over one million people, and the borders between these two cities are invisible in the structure of the agglomeration.

The consistency score for Cronbach’s LAP-r Alpha scale was 0.73 (Supplementary Table S1). Strong intercorrelations were observed between all dimensions of the scale, excluding acceptance of death (DA) and the remaining dimensions of the LAP-r scale (Supplementary Table S2).

Each of the subjects underwent a psychometric examination using the following tools: Beck’s Depression Inventory (BDI II) in the Polish standardized version developed by the Psychological Test Laboratory of the Polish Psychological Association (22), Life Attitude Profile – Revised (LAP-R) developed by Gary T. Reker in the version standardized and published by the Psychological Test Laboratory of the Polish Psychological Association (23), De Jong Gierveld Loneliness (DJGLS) in the Polish standardized version (24). Additionally, each of the respondents completed a sociodemographic questionnaire of the authors’ construction, which included questions about age, sex, past diseases, hospitalization due to SARS-CoV-2 infection, oxygen therapy.

### 2.2 Statistical analysis

The non-parametric Mann–Whitney U test was used in the study for analyses of continuous variables with a non-normal distribution in two groups, while for more groups (>2) the non-parametric Kruskal Wallis (K-W) test was used. For significant results in the K-W test, a *post-hoc* Dunn test was performed. Statistically significant results were presented using a box-plot. The Spearman rank correlation test was used to assess the relationship between continuous or ordinal variables, and the level of correlation between the variables was assessed using Spearman’s rank correlation coefficient R. In order to assess the internal consistency of the LAP-r scale in the study, Cronbach’s alpha was the measure of intercorrelation between scale dimensions. The normality of distribution for continuous variables was analyzed using the Shapiro Wilk W test. The results for continuous and ordinal variables were presented using medians with quartiles of 25% and quartiles of 75%, while qualitative variables were presented using frequencies and percentages. The significance level for all analyses was *p* < 0.05. The analyses were performed using the STATISTICA version 13.3 statistical software (TIBCO 2022, Poland).

#### 2.2.1 Scales

The Polish adaptation of De Jong Gierveld Loneliness Scale (DJGLS) by Grygiel et al. (24), developed with the consent of the author of the tool (Cronbach’s alpha 0.89) was applied in the assessment of the feeling of loneliness (24–26). It consists of 11 items, with a five-point score scale for each of them. The higher total DJGLS score reflects a more severe feeling of loneliness (24). The demographic data such as the patients’ age, gender, marital status, residence and education level were collected using a questionnaire. The Polish adaptation of the Life Attitude Profile – Revised (LAP-R) questionnaire (Cronbach’s alpha between 0.70 and 0.80) was used to assess the sense of meaning in life (27, 28). The questionnaire, consisting of 8 scales, originally developed by Gary T. Reker, published by the Psychological Test Laboratory of the Polish Psychological Association. Six scales, including Purpose (life goals and a sense of direction), Coherence (understanding oneself and the environment), Choice/Responsibleness (a view on the ability to make life choices), Death acceptance (no fear of death, accepting death as normal), Existential vacuum (absence of meaning in life, goals and direction), Goal seeking (desire for new experiences) are simple. Each item rating ranges from 1 (strongly disagree) to 7 (strongly agree) and each subscale has 8 items. Except for the Existential vacuum scale, which is scored negatively, all the other scales are scored positively. The two remaining complex scales,. including The Personal Meaning Index (life goals, sense of direction, understanding of oneself and the environment)—a sum of coherence and purpose, and Existential Transcendence (a general measure of life attitudes)—a sum of purpose, coherence, choice/responsibleness, death acceptance with existential vacuum and goal seeking subtraction, are calculated on the basis of the simple scales.

The aim of the use of the Beck Depression Inventory version II (BDI) was to assess the severity of depressive symptoms (or depressiveness). The scale, adapted to Polish, validated and published by the Psychological Test Laboratory of the Polish Psychological Association, consists of 21 items assessing the occurrence and intensity of depressive symptoms within the past 2 weeks. Each item is scored from 0 to 3, and a total score can range from 0 to 63 points. The higher scores indicate the greater the severity of depressiveness.

## 3 Results

**Figure 1:**
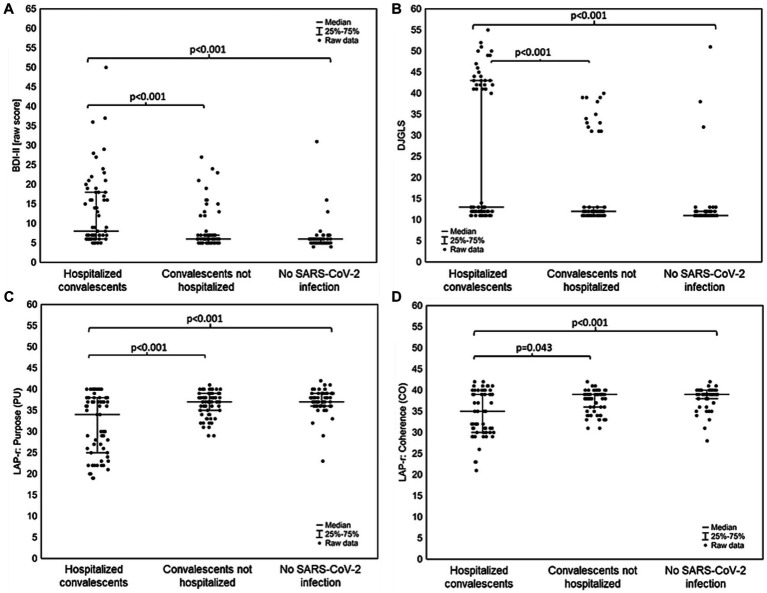
Differences between convalescents who were hospitalized for SARS-CoV-2 infection, convalescents who did not require hospitalization for SARS-CoV-2 infection and people who had never had SARS-CoV-2 infection in **(A)** severity depression according to the BDI-II scale; **(B)** feeling of loneliness on the DJGLS scale; **(C)** dimension of the LAP-r scale: Purpose (CU); **(D)** dimension of the LAP-r scale: Coherence (CO).

**Figure 2:**
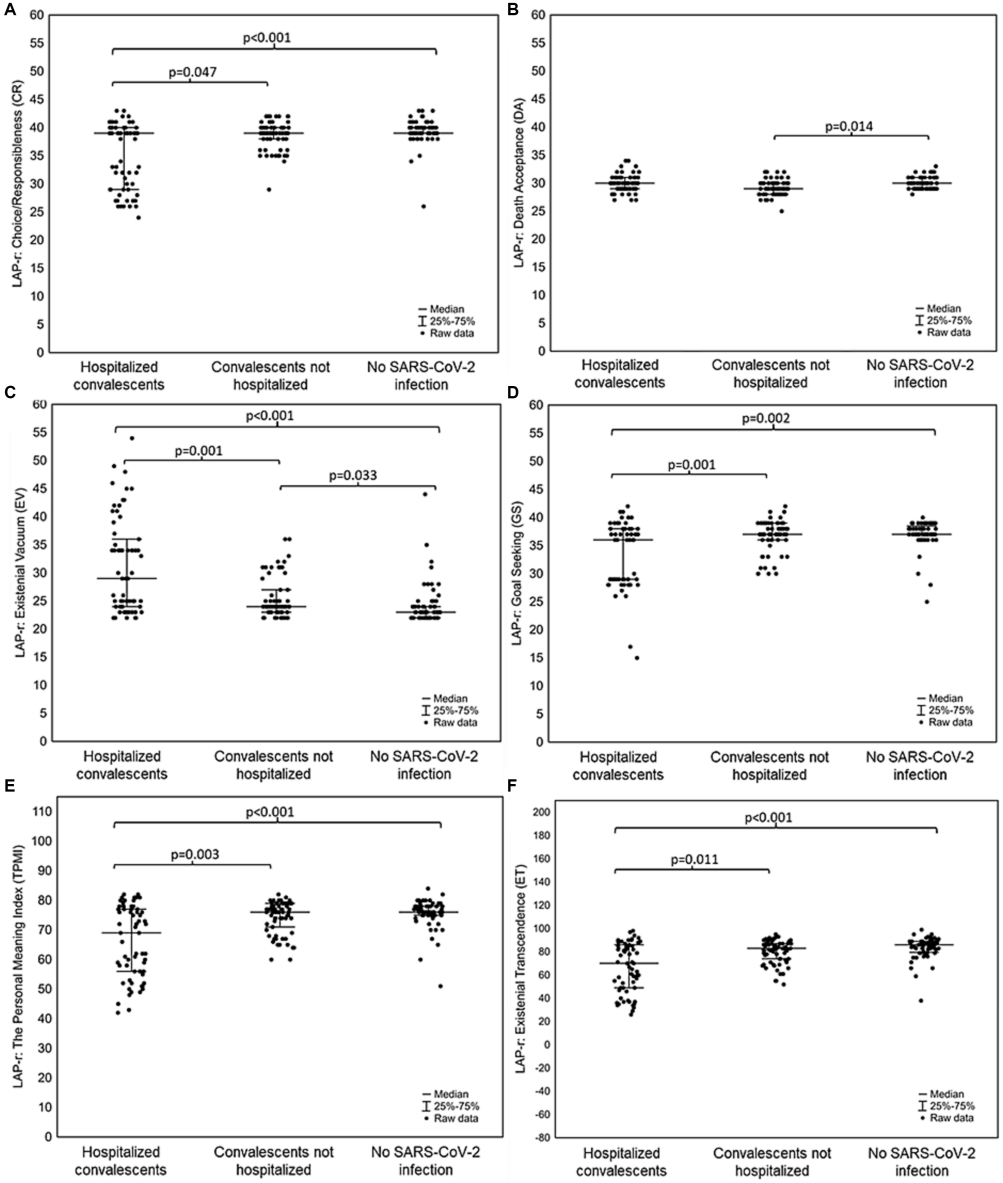
Differences between convalescents who were hospitalized due to SARS-CoV-2 infection, convalescents who did not require hospitalization due to SARS-CoV-2 infection and people who had never had a SARS-CoV-2 infection in **(A)** dimension LAP-r scale: Choice/Responsibleness (CR); **(B)** dimension of the LAP-r scale: Death Acceptance (DA); **(C)** dimension of the LAP-r scale: Existential Vacuum (EV); **(D)** dimension of the LAP-r scale: Goal Seeking (GS); **(E)** dimension of the LAP-r scale: The Personal Meaning Index (TPMI); **(F)** dimension of the LAP-r scale: Existential Transcendence (ET).

In the assessment of differences between hospitalized convalescents, non-hospitalized convalescents and people without SARS-CoV-2 infection, it was shown that convalescent patients who had been hospitalized with SARS-CoV-2 had the highest depression severity score on the BDI-II scale [Median (Q1–Q3): 6.00 (5.00–7.00), *p* < 0.001], and subjects without SARS-CoV-2 infection had the lowest [Median (Q1–Q3): 6.00–5.00–6.00, *p* < 0.001]. Similarly, hospitalized patients scored higher in the severity of the sense of loneliness in the DJGLS scale [Median (Q1–Q3): 13.00 (12.00–43.00)] than the other groups [Median (Q1–Q3)—non-hospitalized convalescents: 12.00 (11.00–13.00), *p* < 0.001, Median (Q1–Q3), subjects without SARS-CoV-2 infection: 11.00 (11.00–12.00), *p* < 0.001]. For LAP-r scale dimensions, convalescents who had been hospitalized for SARS-CoV-2 infection, showed statistically significantly lower results in the following dimensions: Purpose (PU) [Median (Q1–Q3): 34.00 (25.00–38.00)], Coherence (CO) [Median (Q1–Q3): 35.00 (30.00–39.00)], Goal Seeking (GS) [Median (Q1–Q3): 36.00 (29.00–38.00)], The Personal Meaning Index (TPMI) [Median (Q1–Q3): 69.00 (56.00–77.00)] and Existential Transcendence (ET) [Median (Q1–Q3): 70.00 (49.00–86.00)] than the other study groups, and a statistically significantly higher score in the Existential Vacuum (EV) dimension [Median (Q1–Q3): 29.00 (24.00–36.00)] than the other groups. In the case of the dimension of the LAP-r scale concerning the Death Acceptance (DA), there were no statistically significant differences between convalescents hospitalized and non-hospitalized due to SARS-CoV-2 infection, while statistically significant differences were found between convalescent patients who did not require hospitalization and patients who had never had a SARS-CoV-2 infection [Median (Q1–Q3): 29:00 (28:00–30:00) vs. 30:00 (29:00–31:00), *p* = 0.014]. The results are shown in Figures 1, 2.

**Figure 3:**
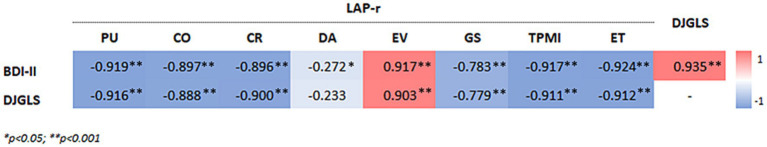
The relationship between the severity of depression on the BDI-II scale and the sense of loneliness on the DJGLS scale and the dimensions on the LAP-r scale, as well as the relationship between the sense of loneliness on the DJGLS scale and the dimensions on the LAP-r scale for convalescent patients who were hospitalized for SARS-CoV-2 infection.

In the assessment of the relationship between the severity of depression and the sense of loneliness, and in the dimensions of the LAP-r scale, convalescent patients who had been hospitalized due to SARS-CoV-2 infection obtained the strongest, statistically significant correlations (Figure 3). In patients in this group, a very strong, statistically significant, positive correlation was found between the feeling of existential vacuum and the severity of depression (*R* = 0.917, *p* < 0.001), as well as between EV and the sense of loneliness in the DJGLS scale (*R* = 0.903, *p* < 0.001). Very strong, negative, statistically significant correlations were also obtained between PU, TMPI and ET and the severity of depression on the BDI-II scale and the sense of loneliness on the DJGLS scale.

**Figure 4:**
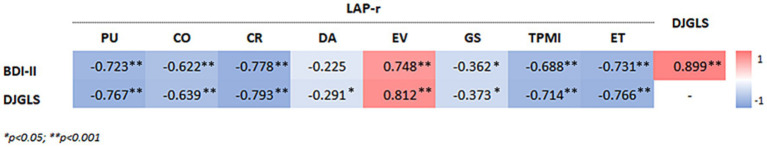
Relationship between the severity of depression in the BDI-II scale and the feeling of loneliness in the DJGLS scale and the dimensions in the LAP-r scale, as well as the relationship between the sense of loneliness in the DJGLS scale and the dimensions in the LAP-r scale for convalescent patients who did not require hospitalization with due to SARS-CoV-2 infection.

In the case of convalescents who did not require hospitalization due to SARS-CoV-2 infection (Figure 4), medium but statistically significant correlations were observed, excluding the dimension of death acceptance on the LAP-r scale and the severity of depression on the BDI-II scale (*R* = 0.225, *p* = 0.067). Strong, positive, statistically significant correlations were found between PE and BDI-II (*R* = 0.748, *p* < 0.001) and between PE and DJGLS (*R* = 0.812, *p* < 0.001).

**Figure 5:**
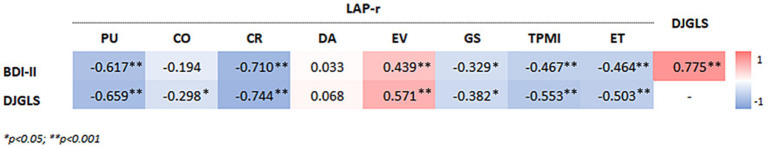
The relationship between the severity of depression in the BDI-II scale and the feeling of loneliness in the DJGLS scale and the dimensions in the LAP-r scale, as well as the relationship between the sense of loneliness in the DJGLS scale and the dimensions in the LAP-r scale for patients who have never had a SARS-CoV-2.

Similar results as in the group of non-hospitalized patients were obtained for patients who did not have SARS-CoV-2 infection (Figure 5). In this case, too, there was no statistically significant relationship between the severity of depression and the dimension of acceptance of death in the LAP-r scale (*R* = 0.033, *p* = 0.802), and additionally no significant correlation was found between the severity of depression and coherence (*R* = −0.194, *p* = 0.137). The strongest, negative, statistically significant correlations were found between Choice/Responsibleness (CR) and BDI-II (*R* = −0.710, *p* < 0.001) and CR and DJGLS (*R* = −0.744, *p* < 0.001).

In all study groups, strong, statistically significant, positive correlations were found between the severity of depression on the BDI-II scale and the sense of loneliness on the DJGLS scale, where the strongest correlation was shown for convalescent patients who had required hospitalization due to SARS-CoV-2 (*R* = 0.935, *p* < 0.001) (Figure 3), and a weaker, but still strong, correlation was shown in patients who did not have SARS-CoV-2 infection (*R* = 0.775, *p* < 0.001) (Figure 5).

## 4 Discussion

Our results are one of the first in the world studies of this kind on loneliness and a sense of meaning in life in post-Covid convalescents. Hospitalization in the intensive care unit, a life-threatening condition, oxygen therapy and sometimes intubation, as well as the atmosphere around the pandemic, were a very strong stressor that could trigger a reaction to severe stress in the form of adaptive syndrome or post-traumatic stress disorder (29). These syndromes, in the absence of adequate support in a widespread atmosphere of danger, led in a simple way to anxiety and mood disorders and a feeling of loneliness (30). During a pandemic, an infected person automatically becomes stigmatized and excluded, which may further exacerbate the studied variables (31). Experiencing a pandemic is a new phenomenon, unknown to science for a hundred years, requiring reliable research in the field of social psychology, clinical psychology and sociology (32). The global pandemic has created a global sense of existential crisis, which at the level of the individual requires a reformulation of concepts such as life, death, worldview or sense of meaning in life. It would be worth getting to know the determinants and intermediary variables regarding the mental functioning of seriously ill people in the pandemic. Such a disease differs quantitatively and qualitatively as well as contextually from a severe disease that can affect anybody outside the pandemic period. An interesting observation from our research is the fact that people who have been hospitalized have lower dimensions of the sense of meaning in life. The above probably means that SARS-CoV-2, hospitalization and a sense of danger are the cause of the breakdown of the life line in these subjects. It manifests itself not only in depression and a feeling of loneliness, but above all in the loss of life goals and existential vacuum. The problem can also be looked at from a different perspective. People with a vaguely formulated sense of meaning in life, depressed and lonely may possibly be more susceptible to a severe course of the disease. It is known from health psychology that personal stress coping resources are an important element of resilience and resistance to illness (33). The personal resources for coping with stress and difficult situations play a key role in the incidence of numerous diseases, their course and prognosis (33). Our study will not determine whether depression, loneliness and a low sense of meaning in life in the case of hospitalized people with SARS-CoV-2 are primary—the lack of resources as the cause of the disease—or secondary (severe course of the disease as the cause of depression, loneliness and low sense of meaning in life). The answer to this question requires many years of follow-up and prospective cohort studies in anticipation of the development of the next pandemics. Such research would be worth conducting, especially since the influence of the psyche on resistance to diseases is discussed more and more frequently. Psychoneuroimmunology dealing with this issue is a subdiscipline from the borderline of medicine and psychology (34, 35).

In subjects after hospitalization, the dimension of existential vacuum correlated positively with depression and a sense of loneliness. All three variables, in our opinion, are the result of both severe illness and the situation in which patients find themselves. As observed in the hospitalized group, the dimensions of purpose, personal meaning and balance of life attitudes correlated negatively with the severity of depression. In the group of people who had been infected with the SARS-CoV-2 virus without hospitalization, the correlations were similar to those in the group of hospitalized people. It is interesting that in this group of respondents, as in the group of hospitalized patients, a positive correlation of the intensity of existential vacuum with the severity of depression and the intensification of the sense of loneliness was observed. Therefore, it should be cautiously concluded that the structure of the psyche of the examined people defined in the form of a network of correlations in both groups is similar, and the differences are quantitative only. This phenomenon can be explained on the one hand by a similar psychological profile of people who have had the SARS-CoV-2 infection. On the other hand, quantitative differences can be explained by the severity of the course and the form of treatment (hospitalization vs. staying at home). The people who have never been infected with the SARS-CoV-2 virus had a different psychical structure. In this group, the acceptance of death did not correlate with depression, and the intensity of the life control dimension correlated negatively with depression and the feeling of loneliness. It can therefore be suggested cautiously that the psychological profile of people who have had SARS-CoV-2 and the profile of people who have not contracted SARS-CoV-2 is to some extent different. The above thesis requires empirical verification. The analogous phenomenon of the relationship between the psychological profile and the incidence of certain diseases is widely known in clinical psychology. For instance, the type A (36–38) and D (39) behavior patterns predispose to the development of cardiovascular diseases, including primarily ischemic heart disease (40). In turn, cancer is more common in people who are conciliatory and suppress their aggression (41). Based on the analysis of the results obtained by us regarding the SARS-CoV-2 convalescents, we postulate the existence of a specific psychological profile conducive to susceptibility to the coronavirus infection. Among the numerous works on COVID-19, there are no publications referring to the correlations between the personality structure and susceptibility to the disease. On the other hand, the psychological effects of SARS-CoV-2 infection and the impact of the pandemic on the mental health of caregivers of patients are widely discussed (42, 43). Our results are consistent with those of the papers published in the recent years, which also demonstrated an increase in loneliness and depressive symptoms during the COVID-19 pandemic (16, 17).

The question concerning the treatment options for anxiety and depression as well as the increased sense of loneliness in the course of the pandemic should be asked. In addition to antidepressants, it seems that cognitive behavioral therapy and mindfulness-based cognitive behavioral therapy should be crucial. This is all the more justified because the cause of the disorders is an exogenous factor (isolation, anxiety, the atmosphere accompanying the pandemic), and not endogenous, as in the typical course of unipolar or bipolar affective disorder (44, 45).

### 4.1 Limitations of the study

Our study is more of a pilot study than a population study in character. The results obtained by us and the correlations described should be replicated by other researchers on a larger sample of people. Aware of these limitations, we have drawn quite cautious conclusions concerning the results of the study.

The study is based on two centers and applies only to the population of Central Poland. The results obtained by us might have been different if they had been performed on a different population with a different genetic heritage and living in different than Polish cultural conditions.

In the control group, it was not checked whether the persons qualified for it had had an asymptomatic or unnoticed infection with the SARS-CoV-2 virus. There are also no data on the percentage of people vaccinated against SARS-CoV-2 infection in the particular study groups.

The analysis did not take into account such variables as the presence of chronic complications after infection with the SARS-CoV-2 virus – such as memory disorders, chronic fatigue syndrome, complications like thrombosis, pulmonary fibrosis or others. However, taking into consideration so many variables would require much larger study groups with the numbers of subjects calculated from the statistical model.

## Data availability statement

The original contributions presented in the study are included in the article/supplementary material, further inquiries can be directed to the corresponding author.

## Ethics statement

The studies involving humans were approved by The Bioethics Committee of the Medical University of Lodz–consent no. RNN/137/22/KE, Poland. The studies were conducted in accordance with the local legislation and institutional requirements. The participants provided their written informed consent to participate in this study.

## Author contributions

KS: Writing – original draft, Conceptualization, Data curation, Formal analysis, Investigation, Methodology, Project administration, Resources, Software, Validation, Visualization. TP: Funding acquisition, Supervision, Writing – review & editing. AM: Funding acquisition, Supervision, Writing – review & editing. MS: Writing – review & editing. MR: Writing – review & editing. KK: Writing – review & editing. IS: Writing – review & editing. MK: Supervision, Visualization, Writing – review & editing.
