# Supplementary material for: The sense of loneliness and meaning in life in post-COVID convalescents—a preliminary study
Source: Front Psychiatry. 2023 Dec 21;14:1296385. doi: 10.3389/fpsyt.2023.1296385 (PMC10768000; doi:10.3389/fpsyt.2023.1296385)
Supplement: Supplementary file 1 [file Table_1.DOCX]

Supplementary Material

**Table S1.** Internal consistency for LAP-r scale dimensions

| **LAP-R** | **Mean±SD** | **Median (Q1-Q3)** | **Cronbach's Alpha** | **Correlation** |
| --- | --- | --- | --- | --- |
| Purpose; PU | 35.16±5.31 | 37.00 (34.00-39.00) | 0.62 | 0.98 |
| Coherence; CO | 36.81±4.03 | 39.00 (35.00-39.00) | 0.65 | 0.94 |
| Choice/Responsibleness; CR | 37.63±4.37 | 39.00 (38.00-40.00) | 0.65 | 0.94 |
| Death Acceptance; DA | 29.67±1.43 | 30.00 (29.00-31.00) | 0.74 | 0.18 |
| Existenial Vacuum; EV | 27.00±6.43 | 24.00 (23.00-30.00) | 0.88 | -0.96 |
| Goal Seeking; GS | 35.63±4.38 | 37.00 (36.00-38.00) | 0.67 | 0.77 |
| The Personal Meaning Index; TPMI | 71.97±9.16 | 76.00 (68.00-78.00) | 0.55 | 0.98 |
| Existenial Transcendence; ET | 76.65±16.16 | 83.00 (70.00-88.00) | 0.57 | 0.96 |
| **Summary** |  |  | 0.73 | 0.49 |

**Table S2.** Intercorrelations between dimensions in the LAP-r scale

|  | **PU** | **CO** | **CR** | **DA** | **EV** | **GS** | **TPMI** | **ET** |
| --- | --- | --- | --- | --- | --- | --- | --- | --- |
| **PU** | 1.00 |  |  |  |  |  |  |  |
| **CO** | **0.921 | 1.00 |  |  |  |  |  |  |
| **CR** | **0.936 | **0.864 | 1.00 |  |  |  |  |  |
| **DA** | 0.128 | **0.104 | **0.180 | 1.00 |  |  |  |  |
| **EV** | **-0.950 | **-0.919 | **-0.910 | -0.091 | 1.00 |  |  |  |
| **GS** | **0.818 | **0.743 | **0.835 | 0.133 | **-0.817 | 1.00 |  |  |
| **TPMI** | **0.985 | **0.974 | **0.923 | 0.120 | **-0.955 | **0.801 | 1.00 |  |
| **ET** | **0.970 | **0.950 | **0.936 | *0.203 | **-0.963 | **0.739 | **0.980 | 1.00 |

**p<0.05 (two-sided), **p<0.001 (two-sided)*
